# Nutritional Risk Screening in Cancer Patients: The First Step Toward Better Clinical Outcome

**DOI:** 10.3389/fnut.2021.603936

**Published:** 2021-04-07

**Authors:** Emilie Reber, Katja A. Schönenberger, Maria F. Vasiloglou, Zeno Stanga

**Affiliations:** ^1^Department of Diabetes, Endocrinology, Nutritional Medicine and Metabolism, Bern University Hospital, Inselspital, University of Bern, Bern, Switzerland; ^2^Artificial Organ (ARTORG) Centre for Biomedical Engineering Research, University of Bern, Bern, Switzerland

**Keywords:** cancer, NRS 2002, malnutrition (MeSH), oncology, nutritional screening

## Abstract

Disease-related malnutrition is highly prevalent among cancer patients, with 40–80% suffering from it during the course of their disease. Malnutrition is associated with numerous negative outcomes such as: longer hospital stays, increased morbidity and mortality rates, delayed wound healing, as well as decreased muscle function, autonomy and quality of life. In cancer patients, malnutrition negatively affects treatment tolerance (including anti-cancer drugs, surgery, chemo- and radiotherapy), increases side effects, causes adverse reactions, treatment interruptions, postoperative complications and higher readmission rates. Conversely, anti-cancer treatments are also known to affect body composition and impair nutritional status. Tailoring early nutritional therapy to patients' needs has been shown to prevent, treat and limit the negative consequences of malnutrition and is likely to improve overall prognosis. As the optimisation of treatment outcomes is top priority and evidence for nutritional therapy is growing, it is increasingly recognized as a significant intervention and an autonomous component of multimodal cancer care. The proactive implementation of nutritional screening and assessment is essential for patients suffering from cancer - given the interaction of clinical, metabolic, pharmacological factors with systemic inflammation; and suppressed appetite with accelerated muscle protein catabolism. At the same time, a nutritional care plan must be established, and adequate individualized nutritional intervention started rapidly. Screening tools for nutritional risk should be validated, standardized, non-invasive, quick and easy-to-use in daily clinical practice. Such tools must be able to identify patients who are already malnourished, as well as those at risk for malnutrition, in order to prevent or treat malnutrition and reduce negative outcomes. This review investigates the predictive value of commonly used screening tools, as well as the sensitivity and specificity of their individual components for improving clinical outcomes in oncologic populations. Healthcare professionals' awareness of malnutrition in cancer patients and the pertinence of early nutritional screening must be raised in order to plan the best possible intervention and follow-up during the patients' ordeal with the disease.

## Introduction

Disease-related malnutrition (DRM) is highly prevalent among cancer patients with 40–80% suffering from it during the course of their disease. Factors influencing DRM include among others the type of cancer, the stage, location and nature of treatment (1–3). DRM is a subacute and chronic condition resulting from a deficit in energy, protein, and micronutrient intake resulting in changes in body composition and reduced body function which in turn negatively impact clinical outcome (4). The European Society for Clinical Nutrition and Metabolism (ESPEN) defines a cancer patient as “a patient with a cancer diagnosis who is either waiting for or on cancer-directed treatment, on symptomatic treatment, and/or receiving palliative care” (5). They are consequently in different conditions at treatment start (e.g., normal weight, overweight or obese), undergoing various oncological treatments and reacting to them in a different manner. Many cancer patients experience decreased physiological and biological function, malnutrition, weight gain/loss, fatigue, and psychological distress. Furthermore, many patients experience metabolic changes and a systemic cytokine-related inflammatory process followed by insulin resistance. This metabolic state is associated with reduced appetite (anorexia), increased muscle protein catabolism, and impaired body function. All these factors may further worsen DRM and potentially result in a multifactor wasting syndrome defined as cachexia. It is therefore essential, as part of an adequate multifaceted management regime, to identify and treat patients at nutritional risk in the early reversible cachectic phase before refractory cachexia occurs (6).

Unintentional weight loss is a major problem that impairs body function, survival outcomes and quality of life (6, 7). Unintentional weight loss >5% is experienced in a large proportion of patients with gastric cancer (67%), pancreatic cancer (54%) and lung cancer (35%), thus being the cancer types where malnutrition is very prevalent (1, 8–10). DRM is a common issue in the inhospital setting (32–34%) and the outpatient setting (39%) (1, 11, 12). Approximately 20% of cancer patients die from the consequences of DRM, rather than from the primary disease itself (13, 14). Usually, DRM cannot completely be reversed with a conventional diet and requires artificial nutritional therapy. In an advanced stage when refractory cancer cachexia occurs, the risks and burden of such therapy possibly outweigh the potential benefit (6). DRM has negative effects on health as a whole and is associated with numerous negative outcomes such as increased morbidity and mortality rates, longer hospital stays, delayed wound healing, as well as decreases in muscle function, autonomy and quality of life (15). In this population, DRM negatively affects treatment tolerance (including anti-cancer drugs, surgery, chemo- and radiotherapy), increases side effects, and causes adverse reactions, treatment interruptions and postoperative complications. In addition, anti-cancer treatments are also known to affect body composition and nutritional state. Early nutritional therapy tailored to patients' needs has been shown to prevent, treat, and limit the negative consequences of DRM and may improve prognosis (16). As evidence for the effectiveness of nutritional intervention is growing, it should progressively become a significant part of the multimodal cancer care.

As a first step in the nutritional management of oncologic patients, the ESPEN recommends using a validated screening instrument to assess the nutritional risk for both in- and outpatients (5, 17–19). Screening tools for nutritional risk should be validated by randomized controlled trials, standardized, quick, and easy to use in daily clinical work. As already mentioned, such tools must also be able to identify malnourished or at-risk patients early on in order to prevent and treat malnutrition and reduce negative outcomes. Although many validated screening tools are available and applicable for both oncologic in- and outpatients, there is no current gold standard to detect the risk of DRM. None of the tools performs well enough to consistently establish patients' nutritional status, and no screening or assessment tool on its own is capable of adequate nutrition screening as well as predicting poor nutrition related outcome (20).

Although international nutrition societies agree on the necessity of systematic nutritional screening, it is not an integrated part of standard care in most institutions (17, 21–23). Studies have shown that without such procedures, over 50% of malnourished patients are not identified as at nutritional risk or malnourished and remain untreated (24–26). In one French study, 55% of patients reported reduced food intake after receiving a cancer diagnosis, independent of their nutritional risk category. Nutrition counseling was provided to only 41.4% of those patients (26). In another French study, only 35.8% received nutrition counseling, provided by dietitians (56.3%), hospital practitioners (31.9%), or general practitioners (12.9%) amongst others (27).

There is urgent need to raise oncologists' awareness of the need for early nutritional screening in cancer patients and the necessity for providing rapid, individualized nutritional intervention to reduce risk and severity of malnutrition which could be detrimental to other clinical outcomes such as survival and quality of life (28). Timely screening and prompt identification of nutritional risk facilitates referral to a dietician for nutrition management and leads to improved outcomes (29). An Italian study demonstrated that clinicians can be trained effectively to perform assessments identifying malnutrition and its risks (3). An integrated nutritional screening would help identify nutritional risk which must then be addressed using a multidisciplinary approach. Clinical team members must be aware which care setting, population, and age group a tool was developed for before implementing any given specific nutritional screening in their institution (29).

The aim of this review is to present an overview of validated nutritional screening tools, which enable quick identification, therapy, and better outcome in oncological patients. It was therefore designed to investigate the predictive value of commonly used screening tools, as well as and the sensitivity and specificity of their individual components regarding the improvement of clinical outcomes in cancer patients.

## Requirements and Purpose of Nutritional Screening

The ESPEN guideline for screening states that “the purpose of nutritional screening is to predict the probability of a better or worse outcome due to nutritional factors, and whether nutritional treatment is likely to influence this” (17). Outcomes may therefore be defined as (i) the maintenance and/or improvement of mental and physical function, (ii) the reduction of treatment- and disease-related complications and their severity, (iii) enhanced recovery, (iv) lower consumption of resources, e.g., length of hospitalization.

A nutritional screening tool must detect the risk of malnutrition, and/or predict whether it is likely to develop or worsen under the present (and future) condition of the patient. It should identify at-risk patients who are likely to benefit from a consecutive nutritional intervention (sensitivity, predictive validity). It should therefore include all parameters relevant to the problem (content validity) and show low interrater variability (reliability). Nutritional screening must assess four principles: (i) the current condition, (ii) its stability (recent involuntary weight loss), (iii) potential for worsening (reduced food intake) and (iv) the negative influence of the disease (stress metabolism associated with severe disease). Using the body mass index (BMI) to define nutritional risk is not reliable - in particular in cancer patients - as other overweight patients who lose weight during treatment would not be considered as at-risk patients, and assessment of sarcopenic patients may be biased (21, 30). Each of these parameters must be scored, providing risk quantification and a direct link to subsequent intervention. An ideal screening tool must be easy to conduct, rapid, non-invasive, not necessitate any calculations or laboratory data, easily interpretable, reproducible and inexpensive (21, 31).

According to the systematic review of van Bockhorst-de van der Schueren et al., thirty-two screening tools have been developed to assess patient nutritional risk (20). Of those tools, twenty-four aimed to assess patient nutritional status (identification of patients likely to benefit from nutritional support), four aimed to predict clinical outcome (complications, morbidity, length of hospital stay and mortality) and four aimed to do both. Other additional tools have been designed for specific populations and care settings. There is currently no “gold standard” among the screening tools for malnutrition. They have mostly been developed using full expert nutritional assessments as a reference. They have also been validated by comparing varying combinations. This has, as a result, meant that different tools are applied in different populations and different settings, and are yielding confusing results.

ESPEN guidelines on nutrition in cancer patients underline the utmost importance of early nutritional screening as the first step of nutrition management - ideally as soon as the cancer diagnosis is made (5, 19). A thorough nutritional assessment and consecutive care plan should be implemented based on the patient's level of risk. The Oncology Evidence-Based Nutrition Practice Guideline for Adults recommends that each patient should be screened for nutritional risk at entry in the oncology clinic. Screening should be routinely repreated throughout the treatment (29). Lastly, screening should initiate a specific action protocol. At-risk patients should ideally be referred to a trained dietician (nutritional consultation), whose comprehensive in-depth nutritional assessment would then be used to tailor an individualized nutritional care plan.

## Recommended Tools for Nutritional Screening

Several screening tools are available; each with its own individual characteristics. ESPEN guidelines for cancer patients recommend the use of the following four in cancer patients: Nutritional Risk Screening 2002 (NRS 2002), Malnutrition Universal Screening Tool (MUST) Mini Nutrition Assessment (MNA) and Malnutrition Screening Tool (MST) (5, 19). The Academy of Nutrition and Diet recommends the use of MST and MUST (29).

All these tools will be briefly presented below, in addition to the Subjective Global Assessment (SGA) and the Nutriscore. Table 1 summarizes the criteria used in each screening tool.

**Table 1:** Criteria used for the nutritional screening in different tools, modified according to (2, 32).

**Criteria**	**Nutritional screening tools**						
	**NRS 2002**	**MNA**	**MUST**	**MST**	**SGA**	**PG-SGA**	**Nutriscore**
Unintentional weight loss	x	x	x	x	x	x	x
BMI	x	x	x				
Appetite		x		x	x		
Food intake	x	x	x	x	x	x	x
Muscle mass/function/mobility		x			x	x	
Disease state	x		x			x	
Age	x						
Neuropsychological aspects		x					

### Nutritional Risk Screening 2002 (NRS 2002)

An ESPEN working group led by Jens Kondrup developed the NRS 2002 in 2003 (18). It is recommended for hospitalized patients and currently used extensively worldwide. The NRS 2002 was developed based on 128 studies showing the effectiveness of nutritional intervention (18). Its purpose is to identify malnourished hospitalized patients who are likely to benefit from nutritional support. The NRS 2002 has been validated in over 100 clinical trials and is practical and quickly performed (2, 3 min) (21, 30). It starts with a pre-screening of four questions. If one is answered with “yes,” a complete screening must be performed. The NRS 2002 is based on impairment of nutritional status (percentage of weight loss, general condition, BMI, and recent food intake), disease severity (stress metabolism), and age. Each category is rated from 0 (normal) to 3 (severe), and an age ≥70 years adds 1 point. Total scores range from 0 to 7 points. Patients with a total score ≥3 classified as “at nutritional risk” could benefit from nutritional support and improved clinical outcome (18).

### Malnutrition Universal Screening Tool (MUST)

The Malnutrition Advisory Group of the British Association for Parenteral and Enteral Nutrition developed MUST in 1992 to identify patients at nutritional risk, and to predict their clinical outcome (33, 34). It is recommended for outpatient screening by the ESPEN Society and has been validated in various care settings and populations (35). It is similar to the NRS 2002 and includes the following criteria: unintentional weight loss, BMI, and food intake (acute disease-related effect inducing a phase of >5 days with no food intake). Each criterion is rated from 0 to 2. All points are added up and patients with an overall score ≥2 are classified as at nutritional risk.

### Mini Nutrition Assessment (MNA)

The MNA was developed to assess the nutritional status in older people who may be frail, living in long-care facilities, or hospitalized (36). It has been validated through independent clinical record assessments by trained physicians, and comprehensive surveys of food intake, biochemical parameters, and anthropometric measurements (37). The MNA includes eighteen items in four categories: anthropometric, general, dietary, and subjective assessment (38). As administering the MNA is time-consuming (15 min), a shorter version with six items has been developed. It retains the accuracy and validity of the full MNA and only takes about 4 min to complete. The final tallied score ranges from 0 to 30 for the full version and 0 to 14 for the short form. Scores of 17–23.5 indicate risk for malnutrition; with <17 indicating malnutrition in the full version and ≤ 11 signaling a risk for malnutrition in the shorter version (39).

### Malnutrition Screening Tool (MST)

Ferguson et al. developed the MST in 1999. It is a quick screening tool, easy to apply, and includes questions on appetite, food intake, and recent weight loss (31). The sum of both categories totals scores ranging from 1 to 5, whereby a ≥2 calls for action. The MST has been well-validated in both in- and outpatient populations (31).

### Subjective Global Assessment (SGA)

The SGA was developed by Detsky et al. in 1987 (40). It identifies patients at nutritional risk and predicts clinical outcomes, and is rather an assessment than a screening tool as it combines medical history with clinical findings. In addition to issues addressed in other screening tools (weight loss, food intake) the SGA also includes: symptoms possibly influencing food intake, functional capacity, physical examination, and the opinion of the clinician in charge. It is easy to learn, efficient, and used in various clinical settings. It does however require training for the clinicians which is quite time-consuming and often perceived as additional workload (41, 42). The SGA targets chronic and advanced cases of malnutrition (43, 44).

The patient-generated SGA (PG-SGA) is the result of items added and changed over time to more specifically meet the needs of cancer patients and involve them directly in the process (13, 45–47). The PG-SGA has been validated in cancer patients and is the most accepted and widely-used screening tool for this population (45, 47–51).

### Nutriscore

The Nutriscore was recently developed for oncology outpatients as an expert consensus from different dietetic and nutrition units from the Catalan Institute of Oncology on the basis of the MST (52). It includes questions to unintentional weight loss. Additionnally, it includes specific oncologic parameters such as tumor location and anti cancer treatment. The sum of all categories ranges from 0 to 11 points, whereas total score ≥5 points calls for action, i.e., referral to a dietician.

## Validation of Screening Tools in the Oncology Population

Validation of screening tools is important as it shows whether the tool is able to detect what it is intended to or not. Table 2 shows the rating for validation results used in this review. Assessing and reporting the validity of a tool within a defined population and care setting is paramount to ensuring its suitability (21). Despite the relatively high number of nutritional screening tools, very few have been validated in oncologic patients. Unfortunately, study results also differ widely, not only for the various tools, but also between studies using the same tools in differing care settings. The true validity of many tools remains unclear due to methodological concerns in the respective studies (53).

**Table 2:** Rating for validation results adapted from (20).

**Good**	**Fair**	**Poor**
Sensitivity AND specificity >80%	Sensitivity OR specificity >80% but both >50%	Sensitivity OR specificity <50%
Kappa >0.6	Kappa 0.4–0.6	Kappa <0.4

Moreover, it may be misleading to validate a screening tool in the oncologic population as there are many different types of cancer types, stages, sites, etc. The type of treatment received as well as the care setting (in - vs. outpatients) may also influence results. Various screening tools include symptoms that potentially affect nutritional status, e.g., fatigue, pain, gastrointestinal symptoms. These symptoms have been shown to be highly specific (91.1%) but rather poorly sensitive (43.3%), leading to under recognition of at-risk patients (31). Ravasco et al. have shown that, in some types of cancer, the impact of nutritional status deterioration and the reduction of food intake may be more relevant than the stage or site (54). Further, many screening tools assess BMI possibly omitting an impaired body composition, as in overweight/obese patients or in patients with ascites and/or oedema in which there is no change in body weight and the BMI is adequate, but there is an important nutritional impairment.

## Validity and Prognostic Value of Various Screening Tools

Table 3 shows the summary of evidence regarding the validation of the presented nutritional screening tools in the oncologic population.

**Table 3:** Evidence for nutritional screening in the oncologic population.

**References**	**Setting**	**Screening tool**	**Reference standard**	**Sample size**	**Prevalence of nutritional risk**	**Sensitivity (%)**	**Specificity (%)**	**Predictive value**	**Validity**
Bauer et al. (45)	Cancer inpatients	PG-SGA	SGA	71	76 SGA	98	82	95 + 93–	Good
Isenring et al. (55)	Cancer outpatients	MST	PG-SGA	50	34 MST 26 PG-SGA	100	92		Good
Ferguson et al. (31)	Cancer outpatients undergoing radiotherapy	MST	SGA	106	11 SGA 28 MST	100	81	40 + 100–	Good
Arribas et al. (52)	Cancer patients	MST/Nutriscore	PG-SGA	394	23 Nutriscore 28 MST 19 PG-SGA	84 MST97 Nutriscore	86 MST 96 Nutriscore	85 +	Good
Boléo-Tomé et al. (56)	Cancer patients referred for radiotherapy	MUST	PG-SGA	450	31 MUST 29 PG-SGA	80	89	87 + 100–	Good
Shaw et al. (57)	Cancer inpatients	MST	PG-SGA	128	71 PG-SGA 52 MST	66	83	91 + 49–	Fair
Vidal-Casariego et al. (58)	Cancer patients	Nutriscore	MUST	93	44 Nutriscore 70 MUST	59	89	93 + 48–	Fair
Read et al. (42)	Cancer patients	MNA	PG-SGA	157	66 MNA 65 PG-SGA	97	54	59 +	Fair
Amaral et al. (25)	Cancer inpatients	MST/MUST	NRS 2002	130	18 MST 44 MUST 29 NRS	48 MST 97 MUST	94 MST 77 MUST		Poor Fair
Bauer et al. (59)	Cancer inpatients	MUST	SGA	65		59	75		Poor
Roulston et al. (60)	Cancer inpatients	MNA/MST/MUST	Nutritional assessment by a dietician	52		92 MNA 29 MUST 79 MST			
Schwegler et al. (12)	Cancer inpatients undergoing colorectal surgery	NRS 2002	–	186	39				
Bozzetti et al. (1)	Cancer outpatients	NRS 2002	–	1000	34				
Gupta et al. (48)	Ovarian cancer patients	SGA	–	132	50				

### SGA

The SGA is the oldest assessment tool and was in fact based on clinical evaluations. It aims to analyse patient nutritional risk and predict postoperative outcomes (61). SGA is most frequently referenced as a predictor of clinical outcomes. Its fair-to-good validity has been demonstrated by correlating objective measurement of nutritional status with clinical classification and with measurements of hospital morbidity (antibiotics use, infections, length of stay) (61). The construct validity of the SGA remains controversial (61). It has been validated in the oncologic outpatient population, showing high sensitivity of 96% and good specificity of 83%; indicating a high degree of interrater agreement (20).

### PG-SGA

The PG-SGA was derived from the SGA for the oncology population and is considered the gold standard for oncology by the Oncology Nutrition Dietetic Practice Group of the American Dietetic Association (56, 62). PG-SGA has been validated in ambulatory oncology settings. It is highly sensitive and specific and is widely used in other care settings (45, 47, 55). It performs well compared to other tools and is therefore used to validate the other screening methods (57). The PG-SGA closely correlates with patient weight loss in the previous 6 months, length of hospital stay, and quality of life (45, 55). Its sensitivity regarding nutritional risk, however, is poor (40, 63). It functions more for assessment than screening and requires time and training.

### MUST

The content validity of the MUST is also ensured as it was developed by a multidisciplinary working group. It is rated as highly reproducible, consistent, and reliable (κ = 0.88–1.00) by health care professionals (34). Predictive validity in the community setting was established based on studies investigating the effect of (semi)starvation on mental and physical function in healthy volunteers. This has also been extended to other care settings, where it has shown fair-to-good validity. The MUST has been specifically validated in cancer patients showing low sensitivity and specificity (1, 4, 54, 59, 60). It demonstrates good predictive validity for clinical outcome (length of hospital stay and mortality), for rate of hospital admissions, and for the number of visits to general practitioners (35).

### Nutriscore

The Nutriscore is a recent score and proven to be rapid, simple and effective in the outpatient setting. It does not perform better than the MUST in the inpatient setting (47, 64).

### MST

The MST was developed based on the SGA for adult inpatients. It shows fair-to-good validity in this setting (31), which has been confirmed by various studies (31, 55, 60). The MST is a poor predictor for clinical outcome. Compared to NRS 2002 and PG-SGA, the MST performs well in cancer outpatients but poorly in cancer inpatients (57). It is however not predictive for the hospital length of stay in those populations (25).

The MST may also be completed by outpatients themselves and results show high intra- (κ = 0.88) and interrater reliability (κ = 0.92) when compared with dieticians (65). The patient-led MST was tested against the SGA, also showing good validity (sensitivity 94% and specificity 86%) (65).

### NRS 2002

The NRS 2002 is known for its high content validity as it was developed by an ESPEN working group and based on the available literature. Its reliability has been validated by a wide range of health care professionals (nurses, dieticians, and physicians, κ = 0.67 and κ = 0.76 among physicians) (18). The predictive validity of the NRS 2002 was demonstrated by retrospectively analyzing 128 randomized controlled trials on nutritional support, where patients at risk according to the NRS 2002 showed a higher likelihood of positive clinical outcome (41). Increasing number of prospective randomized controlled trials investigating the effect of nutritional support in mixed populations (including cancer patients) using the NRS 2002 show improved clinical outcome (reduced length of stay, complications, and mortality) (64, 66). NRS 2002 demonstrates a fair-to-good validity in mixed hospital populations (20, 66). Ravasco et al. found the NRS 2002 total score to be satisfactory in cancer patients with regard to correlations of recognized prognostic factors (such as tumor type, symptoms, or performance status) (54).

### MNA

The MNA was developed for the elderly population and was originally validated by dietician assessment. Validity of the specific contents has not been reported to date, but its reliability was shown to be fair (κ = 0.51) (36). The short form is accepted to be as valid as the full version. The MNA has been studied in all care settings and has a good validity in the elderly in the community, while its validity in hospital settings is poor-to-fair (36–39, 59). There is currently no evidence for the predictive validity of clinical outcome (length of hospital stay, complications, and mortality) in the elderly population. The MNA lacks specificity for cancer patients due to the inclusion of some criteria, e.g., the use of three medications per day or three full meals per day (42), and its usage in the oncologic population is poorly evaluated (21, 42, 50). One study performed in lung cancer patients demonstrated a better predictive and prognostic value of MNA compared to weight loss alone in the initial evaluation of the impaired nutritional state (67).

## Outlook

Future studies should focus on the validation of nutritional screening tools specifically for oncologic patients - especially as oncologic outpatient numbers are growing. There is a need to further investigate the challenges and differences between identifying risk in oncology outpatients (majority) vs. inpatients (few). Bozzetti et al. reported that 32% of cancer outpatients at nutritional risk in their study had not received treatment, even though 14% had an NRS 2002 total score >3 (1).

One German survey of certified oncologic care centers showed that only around 30% of patients received nutritional counseling during their cancer ordeal (regardless of their nutritional status) and in fact, one third of patients screened were actually at nutritional risk. Furthermore, none of the centers had a systematic screening procedure in place and only very few had dieticians available (68). There is an urgent need for action to fill these gaps. The nutritional care process must become an integral part of oncologic outpatient treatment. Even more recent - oncology tailored- plus other ongoing efforts still do not seem to provide us with the solutions, so needed to tackle these prevalent issues in this still under served critical population. There are efforts to provide guidance/indicators which might attempt to reflect/identify malnutrition, as for example the GLIM criteria – a diagnostic and operational framework, and not a measurement tool. One could argue that, ultimately, albeit considering risk definition, it might be worth exploring, as it might well prove suited to better reflect the stratified/personalized nature of oncology, and just might offer an alternative to the traditional approach of a “one size fits all” tool. Future efforts should concentrate on the predictive and prognostic value of each criteria of the screening tools, as it can be expected that all of them will have different weights across such diverse presentations, as it is with oncology- i.e., disease biology, organ, stage, treatment stage, etc. Establishing predictive and prognostic criteria is of the utmost importance in a field like oncology.

## Conclusion

Screening tools are the first step in the nutritional care process. All screening tools presented in this review substantially or moderately agree with each other. Some may help detect nutritional risk, others may predict clinical outcome, others do both in defined populations. There is currently no general screening tool which can predict clinical outcome in every patient group, in all care settings - especially not for the oncologic population mainly due to the heterogeneity of the disease within patient groups and treatment settings. Additionally, the cancer care journey of each patient is unique – the disease itself is very different within each individual patient. The quick and easy screening tools mostly lack sensitivity. High sensitivity may be preferred to higher specificity for screening tools. Patients identified as being at high nutritional risk should undergo further assessment by a dietician and receive an individualized care plan. Health care professionals must be aware of these limitations and implications while screening patients, and must carefully select the appropriate nutritional screening tool depending on the target population, care setting, etc. The validation of screening tools in the oncologic population is inadequate, however, screening is still the most important goal. Consistent integration across the board of the oncology care pathways should be achieved. As malnutrition remains a distressingly undertreated issue, early screening and consecutive rapid initiation with adequate nutritional support should be an integral part of the multimodal oncologic regime – always with the intention of maintaining and improving patients' clinical outcome and quality of life.

## Author Contributions

ER: conception and design and writing–original draft. KS: conception and design and review of the literature. MV: writing–review/editing. ZS: conception and design and writing–review/editing. All authors: contributed to the article and approved the submitted version.

## Conflict of Interest

The authors declare that the research was conducted in the absence of any commercial or financial relationships that could be construed as a potential conflict of interest.
